# Optimization of a suite of flathead catfish (*Pylodictis olivaris*) microsatellite markers for understanding the population genetics of introduced populations in the northeast United States

**DOI:** 10.1186/s13104-021-05725-2

**Published:** 2021-08-16

**Authors:** Shannon L. White, Michael S. Eackles, Tyler Wagner, Megan Schall, Geoff Smith, Julian Avery, David C. Kazyak

**Affiliations:** 1 Natural Systems Analysts, Inc. Under Contract to the U.S. Geological Survey, Eastern Ecological Science Center, Kearneysville, WV 25430 USA; 2 U.S. Geological Survey, Eastern Ecological Science Center, Kearneysville, WV 25430 USA; 3 U.S. Geological Survey, Pennsylvania Cooperative Fish and Wildlife Research Unit, Pennsylvania State University, University Park, PA 16802 USA; 4 Biological Sciences, Pennsylvania State University, Hazleton, PA 18202 USA; 5 Division of Fisheries Management, Pennsylvania Fish and Boat Commission, Bellefonte, PA 16823 USA; 6 Department of Ecosystem Science and Management, Pennsylvania State University, University Park, PA 16802 USA

**Keywords:** Microsatellites, Flathead catfish *Pylodictis olivaris*, Population genetics

## Abstract

**Objective:**

Flathead catfish are rapidly expanding into nonnative waterways throughout the United States. Once established, flathead catfish may cause disruptions to the local ecosystem through consumption and competition with native fishes, including species of conservation concern. Flathead catfish often become a popular sport fish in their introduced range, and so management strategies must frequently balance the need to protect native and naturalized fauna while meeting the desire to maintain or enhance fisheries. However, there are currently few tools available to inform management of invasive flathead catfish (*Pylodictis olivaris*). We describe a suite of microsatellite loci that can be used to characterize population structure, predict invasion history, and assess potential mitigation strategies for flathead catfish.

**Results:**

Our panel of 13 microsatellite loci were polymorphic and appear to be informative for population genetic studies of flathead catfish. We found moderate levels of diversity in four nonnative collections of flathead catfish in the Pennsylvania and Maryland sections of the Susquehanna River and the Schuylkill River, Pennsylvania. Analyses suggested patterns of genetic differentiation within- and among-rivers, highlighting the utility of this marker panel for understanding the structure and assessing the degree of connectivity among flathead catfish populations.

## Introduction

Flathead catfish (*Pylodictis olivaris*) is a large piscivorous catfish native to the Mississippi, Mobile, and Rio Grande basins in the United States that has experienced rapid range expansions following permitted and non-permitted stocking [1]. Once introduced, there are relatively few environmental or intrinsic constraints to dispersal and the species typically experiences high invasion success owing to its long lifespan, fast growth, early maturation, high fecundity, high salinity tolerance, and lack of natural predators [2, 3]. Negative effects of introduced flathead catfish on migratory and resident fishes, including threatened and endangered species, are well-documented [4–6], and models suggest that predation and competition by flathead catfish suppress native fish biomass as much as 50% [7]. Together, high propagule pressure and interactions with native fauna make flathead catfish one of the most harmful of all introduced fishes in the United States [8].

Although introduced flathead catfish populations can have substantial deleterious effects on native biota, popular recreational fisheries have developed wherever they have been introduced. Given the prolific nature of the species, popularity of the fishery, and angler behavior (e.g., catch-and-release fishing), eradication of nonnative flathead catfish may not be feasible or warranted for many populations [9]. There are few resources available to assist in the development of flathead catfish management strategies, and tools are needed to help limit flathead interactions with native and naturalized fish populations while recognizing the growing popularity of the recreational fisheries that have resulted from their introduction. In particular, understanding population genetic structure may improve management outcomes by providing insights into demographic and evolutionary trajectories, spatial scales of metapopulation connectivity, as well as historical and future invasion pathways. Ultimately, this can help identify the most efficacious conservation strategies to use in flathead catfish management, both within the focal areas and in other watersheds which may be facing future invasions.

Here, we describe a panel of microsatellite loci that can be used to support management of flathead catfish populations through studies of population genetics. We highlight the utility of this marker panel in a population genetic analysis of flathead catfish collected from two river basins (Delaware and Susquehanna rivers) within its introduced range in Pennsylvania and Maryland.

## Main text

### Methods

**Fig. 1:**
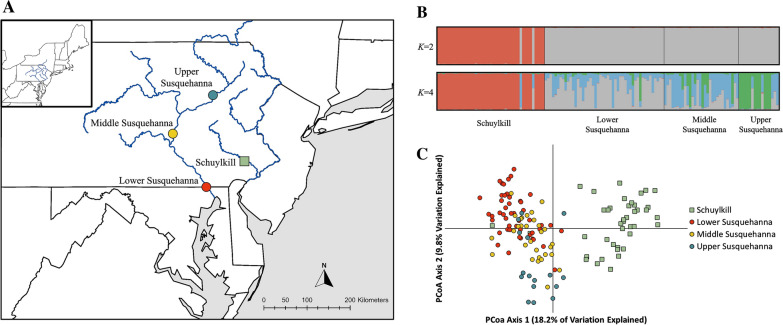
**A** Location of flathead catfish (*Pylodictis olivaris*) samples sites on the Schuylkill and Susquehanna rivers in Pennsylvania and Maryland. **B** Proportion of individual membership to each of *K* = 2 (top) and *K* = 4 (bottom) genetic clusters inferred from STRUCTURE analysis for four collections of flathead catfish. **C** First two dimensions of the principal coordinate analysis (PCoA) for flathead catfish collected from the Schuylkill and Susquehanna rivers. Map produced using ArcGIS

Flathead catfish were collected from one location in the Schuylkill River in the Delaware River Basin in eastern Pennsylvania and three locations distributed along the Susquehanna River in central Pennsylvania and Maryland (Fig. 1a). Fish were collected within a 40-km reach at each sampling location and sampling locations were separated by at least 180 km of hydrologic distance. An approximately 1cm^2^ sample of the caudal fin was collected from each individual, preserved in 95% ethanol, and provided to the U.S. Geological Survey (USGS) Eastern Ecological Science Center, Kearneysville, WV. All fish sampling and tissue collections were conducted in agreement with the Pennsylvania State University Institutional Animal Care and Use Committee (PROTO201901210).

Microsatellite marker panels have not been previously described for flathead catfish; however, studies have suggested that microsatellites are well-conserved among ictalurid catfishes [10]. Therefore, with the goal of establishing a suite of at least 12 polymorphic loci, we created a list of 25 candidate loci from reviewed literature on channel catfish (*Ictalurus punctatus* [10–14]). The candidate loci were selected based on the number of alleles observed in channel catfish, allele size, and repeat motif structure. Additionally, some loci had previously been shown to cross amplify in flathead catfish [10]. Flathead catfish DNA was isolated from tissue samples using the Qiagen Blood and Tissue extraction kit (Qiagen, Valencia, CA, USA) and screened DNA against the 25 candidate loci. Primers were modified to include a 19 bp M13 tag [15] to the 5′ end of each forward marker to facilitate use of the ABI 3500 capillary electrophoresic Genetic Analyzer. All 15 µl PCR reactions were carried out with a third dye-labeled M13 primer complimentary to the tagged forward primer [16].

Of the 25 candidate loci, six loci failed to amplify under varying conditions, including subjection to the 50 °C to 64 °C gradient program on a Bio-Rad T100 thermal cycler. Another six loci proved to be monomorphic in the individuals analyzed. The remaining 13 loci that successfully amplified were ultimately chosen for our final panel based on clean amplification, free of artifacts and any co-amplification, number of alleles observed, and annealing temperature compatibility (Table 1). Each of these 13 markers was re-labeled with a fluorescent dye (FAM, NED, VIC or PET) attached to the 5′ end of the forward primer for detection and fragment length analysis on the ABI 3500 as well as accommodating the multiplexing of multiple loci per reaction. Multiplex configurations were determined with the use of Multiplex Manager 1.0 software [17] resulting in three multiplex reactions or ABI 3500 Genetic Analyzer injections per individual (see Table 1 for details).

**Table 1:** Characteristics of 13 microsatellite loci tested for genetic analyses of flathead catfish (*Pylodictis olivaris*)

Locus	Primer Sequences 5′-3′	Multiplex mix	Fluorescent dye	μM	Size range (bp)	Repeat motif	Source
*GY113J02*	F: CACGTTCAGGCCAATACAACAC	B	6-FAM	0.3	370–400	AAT_(17)_	[11]
	R: GTTTGTCCACTACCTTGTGCCC						
*GY047K03*	F: CCCTCTATGCCTGTGATTGTTTATG	B	6-FAM	0.2	190–230	Not available	[11]
	R: GTTTGTCCACCAAGTCCCTGTGTAAC						
*IpCG0071*	F: CGAAGGTTTATAACTAAGGAGCAGG	B	VIC	0.1	145–190	AGAT_(11)_GGAT_(3)_AGAT_(2)_ GGAT_(6)_[AGAT_(1)_GGAT_(3)_]_4_	[11]
	R: GTTTGTACCTGGCTGTGAAGACAC						
*71–75*	F: CGCTAAATGATCCATTCCACC	C	PET	0.2	110–160	CA_(12)_	[11]
	R: GTTTCAGTGCCGCCATTCTCAC						
*Ip077*	F: GAAACACAATGTACAGTAAGCTG	C	6-FAM	0.2	110–150	GT	[14]
	R: GCTGCTTCTTATGGAATCTC						
*IpCG00189*	F: GATCCTGTGCTAAAGAAACCAAG	A	VIC	0.1	200–300	AAT	[12, 13]
	R: GTGCCGCAGTGTGTTGTAAA						
*Ip271*	F: TGGGGAAAAAGAAAGTAATAACG	A	6-FAM	0.2	155–185	TG_(18)_GA_(19)_	[10]
	R: CAGTAGAGCTTTGAAGCCATTC						
*Ip357*	F: CCTGCCACCATATCAGTGAATTT	A	NED	0.2	100–160	CA_(18)_	[10]
	R: GATAATGAGTCTCCGGAGGTGC						
*Ip365*	F: TAAAGGATCTGATTCACCGTATC	B	PET	0.3	100–145	CA_(13)_	[10]
	R: AAACCGCTAACCTACCCTCT						
*Ip372*	F: GGCACTGAGGTTTGGGCTGCAC	A	PET	0.3	190–220	CA_(8)_	[10]
	R: TGGCATCGCTCCTCATCATCCTG						
*Ip554*	F: GAGATGAAGTGAGATGAAGACAG	A	NED	0.2	220–255	GA_(20)_	[10]
	R: TGCTTAAATAATGACACGGTTC						
*Ip591*	F: CTGCTTTAGGTCCACCCACTGC	C	NED	0.2	130–180	GT, GA	[14]
	R: AGGCACTTGACATTTAGCCTGC						
*IpCG0001*	F: GTACCACTGGTCAGTATCTCC	C	VIC	0.1	175–215	AAT_(20)_	[11]
	R: GTTTCACCATCACCAGAGTCCAGG						

*F* Forward primer, *R*: reverse primer, *Multiplex Mix* assignment of each locus to one of three PCR reactions, *Fluorescent Dye* dye used for each locus within each PCR reaction, *μM* primer concentration used in the PCR reaction, *Size Range (bp)* bp size of the alleles genotyped, *Repeat Motif* motif and number of repeats (in parentheses) for each locus, *Source* citation for the locus

Multiplex PCR’s were generated on a T100 Thermal Cycler (BIO-RAD) in 96-well semi-skirted plates. Each reaction was 15 µl in total volume and contained 1.5 µl genomic DNA (~ 25 ng/µl), 4.6 µl of nuclease-free water, 7.5 µl Qiagen Multiplex 1X MasterMix, 0.2–0.5 µl of each primer (see Table 1 for final concentration for each pair of primers). Cycling conditions included an initial activation and denaturing step of 95 °C for 15 min followed by 34 cycles of 94 °C denaturation for 30 s, 55 °C to 58 °C annealing for 90 s (MP-1 at 58 °C, MP-2 at 55 °C and MP-3 at 56 °C), 72 °C extension step for 60 s and a final extension of 60 °C for 60 s. Upon cycling completion and cooling, 1 µl of each reaction was removed and added to 40 µl of nuclease-free water. From this dilution, 1.5 µl was added to 12 µl ultra-grade formamide and 0.2 µl of GeneScan 500 LIZ size standard. This mixture was briefly centrifuged to remove any bubbles that would impede capillary electrophoresis and then denatured for 5 min at 95 °C, cooled for 5 min then loaded on an 8-capillary ABI-3500 for fragment length analysis. Allele calling and binning was performed with GeneMapper 4.0 software (Applied Biosystems).

#### Data analysis

Final testing of the microsatellite locus panel included *n* = 43 individuals from the Schuylkill River, and *n* = 48, 30, 16 individuals from the lower, middle, and upper Susquehanna River, respectively. Genotypic data were analyzed in MICRO-CHECKER v 2.2.3 [18] to assess the occurrence of null alleles, large allele dropout, and scoring errors.

We used R package GENEPOP [19] to perform exact tests to determine if the distribution of genotypes at each locus conformed to Hardy–Weinberg equilibrium (HWE) and test for linkage disequilibrium (LD). Significance levels for HWE and LD tests were adjusted using the sequential Bonferroni correction. We used GenAlEx [20, 21] to estimate allelic richness (*A*_*R*_), observed (*H*_*O*_) and unbiased expected (*uH*_*E*_) heterozygosity, the number of private alleles (*P*), and the fixation index (*F*) and the program HP-Rare [22] to estimate rarefied allelic richness (*rA*_*R*_; standardized to 40 alleles) for each collection. We also used GenAlEx to perform an analysis of molecular variance (AMOVA) to understand the spatial scale of genetic variation within and between river basins.

To evaluate the extent of genetic differentiation among collections, we calculated pairwise ${F}_{ST}^{^{\prime}}$ in GenAlEx. We also used the Bayesian clustering program STRUCTURE v 2.3.4 [23] to identify population structure among the four collections. We ran 10 independent STRUCTURE runs for each of *K* = 1 to 5 genetic clusters using the admixture model with no a priori information on sample location. Each STRUCTURE run had 200,000 iterations as burn-in, followed by 100,000 replicates of data collection. Results from STRUCTURE were visualized using STRUCTURESelector [24]. We selected the optimal *K* value using the Δ*K* method [25] but also considered other values of *K* that showed biologically relevant patterns in genetic structure. We also completed a principal coordinate analysis (PCoA) in GenAlEx to further visualize genetic variation within and among collections.

### Results and discussion

Across all four collections of flathead catfish, we found few deviations from HWE or LD, no scoring errors, and no evidence of large allele dropout. However, there was possible evidence of null alleles at two loci (*IpCG00189* and *Ip271*). All 13 loci were polymorphic across the four collections (*A*_*R*_ per locus range = 2 to 6) and heterozygosity and allelic richness were moderate in each collection (Table 2). No private alleles were detected in the middle or lower Susquehanna, but we note a larger number of private alleles in the Schuylkill (*n* = 17) relative to those observed in the upper Susquehanna (*n* = 2) collection.

**Table 2:** Population genetics summary for four collections of flathead catfish (*Pylodictis olivaris*)

Site	*N*	*A*_*R*_	*rA*_*R*_	*H*_*O*_	*H*_*E*_	*uH*_*E*_	*F*	*P*	% Poly Loci	HW # Sig Loci	LD # Sig Pairs	Average Pairwise ${F}_{ST}^{^{\prime}}$
Schuylkill	43	3.77	3.51	0.50	0.51	0.51	-0.01	17	100%	1	2	0.31
Lower Susquehanna	48	2.54	2.54	0.49	0.50	0.51	0.03	0	100%	1	0	0.19
Middle Susquehanna	30	2.54	2.54	0.48	0.50	0.51	0.03	0	100%	0	0	0.14
Upper Susquehanna	16	2.62	2.62*	0.60	0.51	0.53	-0.19	2	100%	0	0	0.20

*N*, sample size; *A*_*R*_, allelic richness; *rA*_*R*_, rarefied allelic richness (standardized to 40 alleles); *H*_*O*_, observed heterozygosity; *H*_*E*_, expected heterozygosity; *uH*_*E*_, unbiased expected heterozygosity; *F*, fixation index; *P*, number of private alleles; % Poly Loci, percent of loci that were polymorphic; HW # Sig Loci, number of loci with significant deviation from HWE; LD # Sig Pairs, number of loci pairs with significant linkage disequilibrium; Average pairwise ${F}_{ST}^{^{\prime}}$, average ${F}_{ST}^{^{\prime}}$ across all pairwise comparisons

* Denotes a rarefied allelic richness value standardized to a larger number of alleles (40) than were sampled in the collection (32) and so the value assumes all alleles were present in the sample

All pairwise ${F}_{ST}^{^{\prime}}$ values were statistically significant (*P* < 0.001), with the largest values observed between the collection site on the Schuylkill and all the sites on the Susquehanna River (Table 2). The STRUCTURE analysis showed the most support for *K* = 2 genetic clusters, which generally corresponded to fish collected in the Schuylkill and Susquehanna rivers. However, two individuals captured in the Schuylkill assigned most closely to the Susquehanna cluster. We also evaluated patterns of differentiation at *K* = 4 genetic clusters, which indicated population structure within the Susquehanna River (Fig. 1b). Results of the PCoA supported conclusions generated from STRUCTURE (Fig. 1c). Additionally, results from AMOVA suggested that 13% of variation was explained by differences between the Schuylkill and Susquehanna basins, with only 5% and 2% explained by differences among collections and individuals, respectively. The remaining 80% of variation was contained within individuals.

Overall, our results highlight the utility of this microsatellite panel for population genetic analysis of flathead catfish. While results should be reviewed with appropriate caution given modest sample sizes, they provide insights into the genetic structure of flathead catfish in Pennsylvania and Maryland. Notably, significant differentiation between collections from the Schuylkill and Susquehanna rivers suggests there may be minimal connectivity between the two rivers, and that the patterns of differentiation may reflect strong founder effects and/or genetically distinct source populations. Strong differentiation between the Schuylkill and Susquehanna rivers is not surprising as these two distinct drainages in the Atlantic Slope are only connected by the C&D Canal between the Chesapeake and Delaware bays.

We observed less differentiation among collections from the Susquehanna River; however, there did appear to be some spatial clustering. For example, many individuals from the upper Susquehanna strongly assigned to a single genetic cluster, though admixture among all Susquehanna collection locations was apparent. This finding could support hypotheses that flathead catfish in the upper Susquehanna originated from a different source, and that the once disjunct population is now connected to more downstream population(s) through downstream migration and continued upstream invasion.

While additional study is warranted, this analysis adds to the discussion of the invasion history of flathead catfish in Pennsylvania [26, 27], and indicates that there may be multiple introduction pathways, including the potential for on-going human transfer within and between drainages (as evidence by the two individuals collected from the Schuylkill River that were more genetically similar to fish from the Susquehanna River). Though connectivity was apparent, genetic structure within the Susquehanna River suggests that different reaches may be demographically independent, suggesting regional management strategies may be effective in mitigating population growth and spread. Additionally, it may be possible to use genetic tools to model invasion rate in a riverscape genetics framework [28] and monitor human-mitigated spread of flathead catfish.

## Limitations

We surveyed four collections in our analysis, each of which is thought to have been established for less than three decades. Genotyping additional collections, including collections within the species’ native range and those with longer residence times will help better characterize allelic patterns at each locus and enable more robust conclusions about the origin of nonnative populations. Further investigation will also help resolve whether the null alleles identified in this study are an artifact of small sample sizes and/or nonrandom sampling.

## Data Availability

Microsatellite genotypes are available at USGS ScienceBase (https://doi.org/10.5066/P98U11RG).

## Abbreviations

USGS: U.S. Geological Survey
HWE: Hardy–Weinberg equilibrium
LD: Linkage disequilibrium
*A*_*R*_: Allelic richness
*H*_*O*_: Observed heterozygosity
*uH*_*E*_: Unbiased expected heterozygosity
*P*: Number of private alleles
*F*: Fixation index
*rA*_*R*_: Rarefied allelic richness
PCoA: Principal Coordinates Analysis
